# Bis(2,2′-bipyridine-κ^2^ *N*,*N*′)(3,5-dinitro-2-oxidobenzoato-κ^2^ *O* ^1^,*O* ^2^)cobalt(II)

**DOI:** 10.1107/S1600536808041974

**Published:** 2008-12-17

**Authors:** Chun-Long Zhong, Xiu-Rong Jiang, De-Cai Wen

**Affiliations:** a Department of Chemistry, Longyan University, Longyan, Fujian 364000, People’s Republic of China

## Abstract

In the title compound, [Co(C_7_H_2_N_2_O_7_)(C_10_H_8_N_2_)_2_], the Co^II^ atom is coordinated by four N atoms from two 2,2′-bipyridine ligands and two O atoms from a 3,5-dinitro-2-oxidobenzoate ligand, displaying a distorted octa­hedral coordination geometry. The crystal structure involves C—H⋯O hydrogen bonds between the 2,2′-bipyridine ligands and the carboxyl­ate and NO_2_ groups of the 3,5-dinitro-2-oxidobenzoate ligand.

## Related literature

For general background, see: Lemoine *et al.* (2004 ▶); Wen *et al.* (2007*a* ▶,*b* ▶); Wen & Xie (2007 ▶); Yin *et al.* (2004 ▶). For related structures, see: Wen *et al.* (2007*c* ▶,*d* ▶); Wen & Liu (2007 ▶).
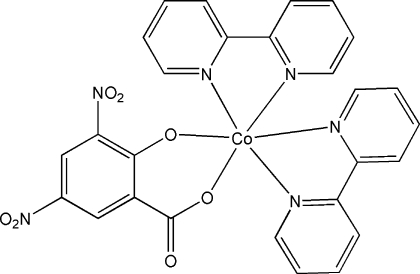


## Experimental

### 

#### Crystal data

[Co(C_7_H_2_N_2_O_7_)(C_10_H_8_N_2_)_2_]
*M* *_r_* = 597.40
Monoclinic, 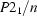

*a* = 8.103 (3) Å
*b* = 21.767 (7) Å
*c* = 14.335 (4) Å
β = 95.804 (13)°
*V* = 2515.4 (15) Å^3^
*Z* = 4
Mo *K*α radiation
μ = 0.74 mm^−1^
*T* = 293 (2) K
0.22 × 0.20 × 0.18 mm

#### Data collection

Rigaku R-AXIS RAPID diffractometer
Absorption correction: none
19627 measured reflections
4427 independent reflections
2952 reflections with *I* > 2σ(*I*)
*R*_int_ = 0.080

#### Refinement

*R*[*F* ^2^ > 2σ(*F* ^2^)] = 0.043
*wR*(*F* ^2^) = 0.090
*S* = 1.02
4427 reflections
370 parameters
H-atom parameters constrained
Δρ_max_ = 0.33 e Å^−3^
Δρ_min_ = −0.31 e Å^−3^

### 

Data collection: *PROCESS-AUTO* (Rigaku, 1998 ▶); cell refinement: *PROCESS-AUTO*; data reduction: *PROCESS-AUTO*; program(s) used to solve structure: *SHELXS97* (Sheldrick, 2008 ▶); program(s) used to refine structure: *SHELXL97* (Sheldrick, 2008 ▶); molecular graphics: *SHELXTL-Plus* (Sheldrick, 2008 ▶); software used to prepare material for publication: *SHELXL97*.

## Supplementary Material

Crystal structure: contains datablocks I, global. DOI: 10.1107/S1600536808041974/hy2168sup1.cif

Structure factors: contains datablocks I. DOI: 10.1107/S1600536808041974/hy2168Isup2.hkl

Additional supplementary materials:  crystallographic information; 3D view; checkCIF report

## Figures and Tables

**Table 1:** Selected bond lengths (Å)

Co1—O2	2.036 (2)
Co1—O3	2.047 (2)
Co1—N5	2.116 (2)
Co1—N3	2.132 (3)
Co1—N4	2.133 (3)
Co1—N6	2.141 (2)

**Table 2:** Hydrogen-bond geometry (Å, °)

*D*—H⋯*A*	*D*—H	H⋯*A*	*D*⋯*A*	*D*—H⋯*A*
C11—H11*A*⋯O1^i^	0.93	2.32	3.166 (5)	151
C21—H21*A*⋯O1^ii^	0.93	2.40	3.275 (4)	157
C16—H16*A*⋯O4^iii^	0.93	2.43	3.171 (5)	137
C25—H25*A*⋯O6^iv^	0.93	2.61	3.209 (5)	123
C9—H9*A*⋯O5^v^	0.93	2.61	3.346 (5)	136

Symmetry codes: (i) 
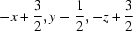
; (ii) 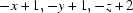; (iii) 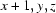; (iv) 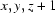; (v) 
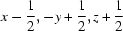
.

**Fig. 1.**
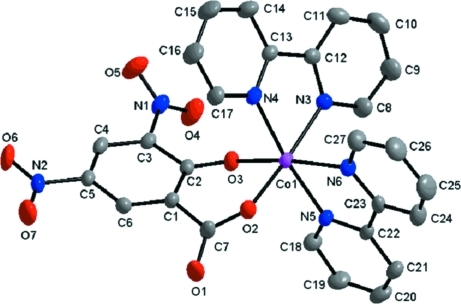
Molecular structure of the title compound, with displacement ellipsoids drawn at the 30% probability level. H atoms have been omitted for clarity.

**Fig. 2.**
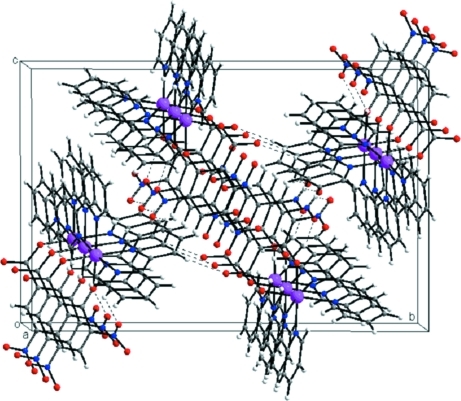
A packing view of the title compound. Hydrogen bonds are represented by dashed lines.

## supplementary crystallographic information

### Comment

Much attention has been paid to metal–salicylate complexes owing to their intriguing structural features and biological applications (Lemoine *et al.*, 2004; Wen *et al.*, 2007a, b; Wen & Xie, 2007; Yin *et al.*, 2004). The assembly process of coordination complexes is highly influenced by lots of factors, such as the structural characteristic of organic ligands, the coordination nature of metal ions, pH value of solution, the metal-to-ligand ratio, counter ions and so on. For example, it has been reported that hydrothermal reactions of Co(CH_3_COO)_2_.4H_2_O, 3,5-dinitro-2-oxidobenzoic acid (3,5-dnsalH_2_) and 2,2'-bipyridine (2,2'-bipy) at different reaction duration give two neutral isomeric metallomacrocycles, Co_4_(2,2'-bipy)_4_(3,5-dnsal)_4_ (Wen *et al.*, 2007c). We report here the structure of a new Co^II^ complex with 3,5-dnsal ligand (Wen & Liu, 2007; Wen *et al.*, 2007d).

The title complex was synthesized under hydrothermal conditions. The Co^II^ atom is coordinated in a distorted octahedral coordination geometry by two O atoms from a 3,5-dnsal ligand and four N atoms from two 2,2'-bipy ligands (Fig.1 and Table 1). The carboxylate group of the 3,5-dnsal ligand is rotated relatively to the aromatic ring with a dihedral angle of 36.8 (2)°. The two pyridyl rings of the 2,2'-bipy ligand containing N5 and N6 are approximately coplanar, exhibiting a dihedral angle of 2.2 (2)°, while the two pyridyl rings of the other 2,2'-bipy containing N3 and N4 deviate from coplanarity with a dihedral angle of 10.6 (2)°. The adjacent mononuclear complex molecules are further connected to each other by C—H···O hydrogen bonds formed between the 2,2'-bipy ligands and the carboxylate and NO_2_ groups of the 3,5-dnsal ligand, resulting in a three-dimensional network structure (Table 2 and Fig. 2).

### Experimental

A mixture of Co(NO_3_)_2_.4H_2_O (0.1 mmol), 2,2'-bipyridine (0.2 mmol), 2-hydroxy-3,5-dinitrobenzoic acid (0.2 mmol) and distilled water (10 ml) was put into a 20 ml Teflon-lined autoclave and then heated at 423 K for 72 h. Brown block-like crystals of the title compound suitable for X-ray analysis were collected from the reaction mixture.

### Refinement

H atoms were positioned geometrically and refined as riding atoms, with C—H = 0.93 Å and *U*_iso_(H) = 1.2*U*_eq_(C).

### Crystal data

**Table:**

[Co(C_7_H_2_N_2_O_7_)(C_10_H_8_N_2_)_2_]	*F*(000) = 1220
*M**_r_* = 597.40	*D*_x_ = 1.578 Mg m^−^^3^
Monoclinic, *P*2_1_/*n*	Mo *K*α radiation, λ = 0.71073 Å
Hall symbol: -P 2yn	Cell parameters from 4427 reflections
*a* = 8.103 (3) Å	θ = 3.0–25.0°
*b* = 21.767 (7) Å	µ = 0.74 mm^−^^1^
*c* = 14.335 (4) Å	*T* = 293 K
β = 95.804 (13)°	Block, brown
*V* = 2515.4 (15) Å^3^	0.22 × 0.20 × 0.18 mm
*Z* = 4	

### Data collection

**Table:**

Rigaku R-AXIS RAPID diffractometer	2952 reflections with *I* > 2σ(*I*)
Radiation source: 18 kW rotating anode	*R*_int_ = 0.080
graphite	θ_max_ = 25.0°, θ_min_ = 3.0°
ω scans	*h* = −9→9
19627 measured reflections	*k* = −25→25
4427 independent reflections	*l* = −17→15

### Refinement

**Table:**

Refinement on *F*^2^	Primary atom site location: structure-invariant direct methods
Least-squares matrix: full	Secondary atom site location: difference Fourier map
*R*[*F*^2^ > 2σ(*F*^2^)] = 0.043	Hydrogen site location: inferred from neighbouring sites
*wR*(*F*^2^) = 0.090	H-atom parameters constrained
*S* = 1.02	*w* = 1/[σ^2^(*F*_o_^2^) + (0.0295*P*)^2^ + 0.2101*P*] where *P* = (*F*_o_^2^ + 2*F*_c_^2^)/3
4427 reflections	(Δ/σ)_max_ = 0.001
370 parameters	Δρ_max_ = 0.33 e Å^−^^3^
0 restraints	Δρ_min_ = −0.31 e Å^−^^3^

### Fractional atomic coordinates and isotropic or equivalent isotropic displacement parameters (Å^2^)

**Table:**

	*x*	*y*	*z*	*U*_iso_*/*U*_eq_	
Co1	0.63835 (5)	0.37028 (2)	0.82451 (3)	0.03493 (13)	
N1	0.4828 (4)	0.32130 (14)	0.51277 (18)	0.0486 (7)	
N2	0.7733 (4)	0.49718 (17)	0.4014 (2)	0.0556 (8)	
N3	0.5787 (3)	0.27604 (12)	0.84364 (17)	0.0406 (6)	
N4	0.8319 (3)	0.32190 (13)	0.76558 (16)	0.0402 (7)	
N5	0.4405 (3)	0.40721 (12)	0.89234 (16)	0.0380 (6)	
N6	0.7350 (3)	0.37120 (13)	0.96914 (16)	0.0406 (6)	
O1	0.6901 (4)	0.54777 (12)	0.73884 (16)	0.0853 (10)	
O2	0.7262 (3)	0.45655 (10)	0.80574 (13)	0.0448 (6)	
O3	0.5155 (3)	0.37447 (11)	0.69267 (13)	0.0433 (5)	
O4	0.3629 (3)	0.30618 (14)	0.55073 (18)	0.0773 (9)	
O5	0.5399 (3)	0.28916 (14)	0.4535 (2)	0.0791 (9)	
O6	0.7632 (4)	0.47298 (14)	0.32404 (17)	0.0844 (9)	
O7	0.8431 (4)	0.54639 (15)	0.41780 (19)	0.0820 (9)	
C1	0.6593 (4)	0.46131 (15)	0.63950 (19)	0.0380 (8)	
C2	0.5739 (3)	0.40396 (15)	0.62656 (19)	0.0346 (7)	
C3	0.5590 (3)	0.38110 (15)	0.53228 (19)	0.0366 (8)	
C4	0.6228 (4)	0.41067 (17)	0.4597 (2)	0.0431 (8)	
H4A	0.6122	0.3937	0.3998	0.052*	
C5	0.7027 (4)	0.46570 (16)	0.4764 (2)	0.0414 (8)	
C6	0.7186 (4)	0.49101 (16)	0.5656 (2)	0.0439 (8)	
H6A	0.7705	0.5289	0.5755	0.053*	
C7	0.6922 (5)	0.49138 (17)	0.7350 (2)	0.0487 (9)	
C8	0.4572 (4)	0.25584 (18)	0.8922 (2)	0.0540 (9)	
H8A	0.3815	0.2843	0.9110	0.065*	
C9	0.4385 (5)	0.19578 (19)	0.9155 (3)	0.0603 (10)	
H9A	0.3536	0.1836	0.9505	0.072*	
C10	0.5475 (5)	0.15383 (18)	0.8863 (3)	0.0616 (11)	
H10A	0.5373	0.1125	0.9011	0.074*	
C11	0.6722 (4)	0.17293 (17)	0.8349 (2)	0.0527 (9)	
H11A	0.7462	0.1447	0.8138	0.063*	
C12	0.6856 (4)	0.23450 (15)	0.8153 (2)	0.0393 (8)	
C13	0.8198 (4)	0.26042 (16)	0.7643 (2)	0.0390 (8)	
C14	0.9282 (5)	0.2248 (2)	0.7189 (3)	0.0665 (11)	
H14A	0.9184	0.1822	0.7182	0.080*	
C15	1.0509 (6)	0.2531 (2)	0.6748 (3)	0.0821 (14)	
H15A	1.1246	0.2298	0.6438	0.098*	
C16	1.0640 (5)	0.3156 (2)	0.6767 (3)	0.0697 (12)	
H16A	1.1474	0.3354	0.6483	0.084*	
C17	0.9511 (4)	0.34852 (18)	0.7214 (2)	0.0502 (9)	
H17A	0.9576	0.3912	0.7211	0.060*	
C18	0.2951 (4)	0.42475 (16)	0.8493 (2)	0.0488 (9)	
H18A	0.2756	0.4195	0.7848	0.059*	
C19	0.1727 (4)	0.45027 (18)	0.8965 (3)	0.0586 (10)	
H19A	0.0719	0.4620	0.8647	0.070*	
C20	0.2032 (5)	0.45792 (19)	0.9916 (3)	0.0638 (11)	
H20A	0.1223	0.4750	1.0252	0.077*	
C21	0.3512 (4)	0.44060 (17)	1.0369 (2)	0.0521 (9)	
H21A	0.3724	0.4457	1.1015	0.063*	
C22	0.4693 (4)	0.41536 (14)	0.98594 (19)	0.0379 (8)	
C23	0.6354 (4)	0.39596 (15)	1.02814 (19)	0.0404 (8)	
C24	0.6875 (5)	0.40303 (18)	1.1225 (2)	0.0591 (10)	
H24A	0.6175	0.4207	1.1626	0.071*	
C25	0.8424 (6)	0.3838 (2)	1.1559 (3)	0.0773 (14)	
H25A	0.8798	0.3890	1.2190	0.093*	
C26	0.9425 (5)	0.3570 (2)	1.0962 (3)	0.0695 (12)	
H26A	1.0473	0.3426	1.1181	0.083*	
C27	0.8848 (4)	0.35174 (17)	1.0030 (2)	0.0538 (10)	
H27A	0.9530	0.3339	0.9621	0.065*	

### Atomic displacement parameters (Å^2^)

**Table:**

	*U*^11^	*U*^22^	*U*^33^	*U*^12^	*U*^13^	*U*^23^
Co1	0.0425 (2)	0.0350 (3)	0.0287 (2)	0.0003 (2)	0.01106 (17)	−0.0013 (2)
N1	0.0501 (17)	0.058 (2)	0.0394 (15)	−0.0070 (15)	0.0118 (14)	−0.0117 (15)
N2	0.074 (2)	0.058 (2)	0.0390 (17)	0.0131 (18)	0.0248 (16)	0.0142 (16)
N3	0.0478 (16)	0.0348 (17)	0.0400 (14)	−0.0024 (13)	0.0090 (13)	−0.0019 (13)
N4	0.0391 (15)	0.0479 (19)	0.0350 (13)	0.0013 (13)	0.0109 (12)	−0.0020 (13)
N5	0.0447 (15)	0.0381 (17)	0.0330 (13)	0.0002 (12)	0.0134 (12)	−0.0013 (12)
N6	0.0463 (16)	0.0409 (17)	0.0352 (13)	−0.0016 (14)	0.0068 (12)	0.0006 (13)
O1	0.181 (3)	0.0328 (17)	0.0465 (14)	−0.0150 (18)	0.0308 (17)	−0.0052 (12)
O2	0.0609 (14)	0.0401 (14)	0.0334 (11)	−0.0088 (12)	0.0056 (11)	−0.0045 (10)
O3	0.0481 (12)	0.0498 (15)	0.0329 (11)	−0.0093 (12)	0.0087 (10)	0.0011 (11)
O4	0.0691 (17)	0.096 (3)	0.0724 (17)	−0.0359 (16)	0.0336 (15)	−0.0251 (16)
O5	0.084 (2)	0.073 (2)	0.0871 (19)	−0.0207 (16)	0.0400 (17)	−0.0407 (17)
O6	0.134 (3)	0.087 (2)	0.0378 (13)	−0.0046 (19)	0.0406 (16)	0.0051 (15)
O7	0.120 (3)	0.065 (2)	0.0671 (17)	−0.0198 (19)	0.0384 (17)	0.0127 (16)
C1	0.0483 (19)	0.036 (2)	0.0308 (15)	0.0044 (16)	0.0119 (14)	0.0000 (14)
C2	0.0349 (17)	0.039 (2)	0.0307 (15)	0.0061 (15)	0.0075 (14)	0.0022 (14)
C3	0.0349 (16)	0.044 (2)	0.0322 (15)	0.0040 (15)	0.0074 (13)	−0.0035 (15)
C4	0.0462 (19)	0.056 (2)	0.0289 (15)	0.0142 (17)	0.0099 (15)	0.0000 (16)
C5	0.050 (2)	0.045 (2)	0.0314 (16)	0.0123 (17)	0.0154 (15)	0.0075 (15)
C6	0.057 (2)	0.036 (2)	0.0407 (18)	0.0039 (16)	0.0148 (16)	0.0030 (15)
C7	0.075 (2)	0.036 (2)	0.0376 (18)	−0.0123 (19)	0.0200 (18)	−0.0043 (17)
C8	0.052 (2)	0.050 (3)	0.064 (2)	−0.0062 (18)	0.0217 (19)	0.0009 (19)
C9	0.057 (2)	0.054 (3)	0.072 (2)	−0.016 (2)	0.017 (2)	0.005 (2)
C10	0.064 (3)	0.040 (2)	0.078 (3)	−0.011 (2)	−0.001 (2)	0.012 (2)
C11	0.052 (2)	0.037 (2)	0.067 (2)	0.0040 (17)	−0.0032 (19)	−0.0035 (19)
C12	0.0463 (19)	0.036 (2)	0.0348 (16)	0.0023 (16)	−0.0007 (15)	−0.0042 (15)
C13	0.0413 (18)	0.042 (2)	0.0342 (16)	0.0051 (16)	0.0073 (15)	−0.0038 (16)
C14	0.082 (3)	0.051 (3)	0.072 (2)	0.012 (2)	0.033 (2)	−0.009 (2)
C15	0.093 (3)	0.071 (3)	0.092 (3)	0.018 (3)	0.057 (3)	−0.008 (3)
C16	0.066 (3)	0.073 (3)	0.077 (3)	0.002 (2)	0.044 (2)	−0.001 (2)
C17	0.052 (2)	0.051 (2)	0.0510 (19)	−0.0008 (17)	0.0215 (18)	0.0010 (17)
C18	0.049 (2)	0.053 (2)	0.0446 (18)	0.0058 (18)	0.0074 (17)	−0.0025 (17)
C19	0.046 (2)	0.060 (3)	0.071 (2)	0.0125 (19)	0.0112 (19)	−0.006 (2)
C20	0.062 (3)	0.060 (3)	0.074 (3)	0.008 (2)	0.033 (2)	−0.015 (2)
C21	0.064 (2)	0.051 (3)	0.0445 (18)	0.0018 (19)	0.0237 (18)	−0.0062 (17)
C22	0.052 (2)	0.0319 (19)	0.0329 (15)	−0.0045 (15)	0.0177 (15)	−0.0032 (14)
C23	0.060 (2)	0.0330 (19)	0.0294 (15)	−0.0070 (16)	0.0102 (16)	0.0016 (14)
C24	0.084 (3)	0.060 (3)	0.0338 (17)	−0.003 (2)	0.0084 (19)	−0.0005 (18)
C25	0.102 (3)	0.087 (4)	0.039 (2)	0.001 (3)	−0.011 (2)	0.005 (2)
C26	0.071 (3)	0.077 (3)	0.056 (2)	0.009 (2)	−0.012 (2)	0.013 (2)
C27	0.051 (2)	0.062 (3)	0.0471 (19)	0.0014 (18)	0.0008 (17)	0.0042 (18)

### Geometric parameters (Å, °)

**Table:**

Co1—O2	2.036 (2)	C8—H8A	0.9300
Co1—O3	2.047 (2)	C9—C10	1.365 (5)
Co1—N5	2.116 (2)	C9—H9A	0.9300
Co1—N3	2.132 (3)	C10—C11	1.373 (5)
Co1—N4	2.133 (3)	C10—H10A	0.9300
Co1—N6	2.141 (2)	C11—C12	1.376 (5)
N1—O4	1.206 (3)	C11—H11A	0.9300
N1—O5	1.227 (3)	C12—C13	1.482 (4)
N1—C3	1.456 (4)	C13—C14	1.382 (5)
N2—O6	1.223 (4)	C14—C15	1.377 (6)
N2—O7	1.223 (4)	C14—H14A	0.9300
N2—C5	1.441 (4)	C15—C16	1.365 (6)
N3—C8	1.336 (4)	C15—H15A	0.9300
N3—C12	1.343 (4)	C16—C17	1.370 (5)
N4—C17	1.339 (4)	C16—H16A	0.9300
N4—C13	1.342 (4)	C17—H17A	0.9300
N5—C18	1.329 (4)	C18—C19	1.373 (5)
N5—C22	1.350 (3)	C18—H18A	0.9300
N6—C27	1.330 (4)	C19—C20	1.371 (5)
N6—C23	1.340 (4)	C19—H19A	0.9300
O1—C7	1.229 (4)	C20—C21	1.359 (5)
O2—C7	1.274 (4)	C20—H20A	0.9300
O3—C2	1.275 (3)	C21—C22	1.376 (4)
C1—C6	1.369 (4)	C21—H21A	0.9300
C1—C2	1.430 (4)	C22—C23	1.480 (4)
C1—C7	1.516 (4)	C23—C24	1.384 (4)
C2—C3	1.434 (4)	C24—C25	1.364 (5)
C3—C4	1.369 (4)	C24—H24A	0.9300
C4—C5	1.371 (5)	C25—C26	1.369 (6)
C4—H4A	0.9300	C25—H25A	0.9300
C5—C6	1.387 (4)	C26—C27	1.375 (5)
C6—H6A	0.9300	C26—H26A	0.9300
C8—C9	1.362 (5)	C27—H27A	0.9300
			
O2—Co1—O3	88.90 (8)	C9—C8—H8A	118.3
O2—Co1—N5	90.10 (10)	C8—C9—C10	118.4 (4)
O3—Co1—N5	95.00 (9)	C8—C9—H9A	120.8
O2—Co1—N3	172.56 (10)	C10—C9—H9A	120.8
O3—Co1—N3	93.79 (9)	C9—C10—C11	119.7 (4)
N5—Co1—N3	96.56 (10)	C9—C10—H10A	120.1
O2—Co1—N4	97.01 (10)	C11—C10—H10A	120.1
O3—Co1—N4	87.98 (9)	C10—C11—C12	118.8 (4)
N5—Co1—N4	172.36 (11)	C10—C11—H11A	120.6
N3—Co1—N4	76.19 (11)	C12—C11—H11A	120.6
O2—Co1—N6	91.19 (9)	N3—C12—C11	121.8 (3)
O3—Co1—N6	171.78 (10)	N3—C12—C13	114.9 (3)
N5—Co1—N6	76.78 (10)	C11—C12—C13	123.3 (3)
N3—Co1—N6	87.14 (10)	N4—C13—C14	121.1 (3)
N4—Co1—N6	100.17 (10)	N4—C13—C12	115.4 (3)
O4—N1—O5	122.3 (3)	C14—C13—C12	123.5 (3)
O4—N1—C3	120.2 (3)	C15—C14—C13	119.2 (4)
O5—N1—C3	117.4 (3)	C15—C14—H14A	120.4
O6—N2—O7	122.6 (3)	C13—C14—H14A	120.4
O6—N2—C5	118.5 (4)	C16—C15—C14	119.7 (4)
O7—N2—C5	118.9 (3)	C16—C15—H15A	120.2
C8—N3—C12	117.9 (3)	C14—C15—H15A	120.2
C8—N3—Co1	124.9 (2)	C15—C16—C17	118.4 (4)
C12—N3—Co1	116.6 (2)	C15—C16—H16A	120.8
C17—N4—C13	118.7 (3)	C17—C16—H16A	120.8
C17—N4—Co1	124.6 (3)	N4—C17—C16	122.8 (4)
C13—N4—Co1	116.2 (2)	N4—C17—H17A	118.6
C18—N5—C22	118.6 (3)	C16—C17—H17A	118.6
C18—N5—Co1	125.0 (2)	N5—C18—C19	122.6 (3)
C22—N5—Co1	116.3 (2)	N5—C18—H18A	118.7
C27—N6—C23	118.9 (3)	C19—C18—H18A	118.7
C27—N6—Co1	125.5 (2)	C20—C19—C18	118.2 (3)
C23—N6—Co1	115.5 (2)	C20—C19—H19A	120.9
C7—O2—Co1	127.1 (2)	C18—C19—H19A	120.9
C2—O3—Co1	121.76 (18)	C21—C20—C19	120.2 (3)
C6—C1—C2	121.0 (3)	C21—C20—H20A	119.9
C6—C1—C7	116.8 (3)	C19—C20—H20A	119.9
C2—C1—C7	122.2 (3)	C20—C21—C22	119.0 (3)
O3—C2—C1	123.7 (3)	C20—C21—H21A	120.5
O3—C2—C3	121.6 (3)	C22—C21—H21A	120.5
C1—C2—C3	114.8 (3)	N5—C22—C21	121.4 (3)
C4—C3—C2	123.5 (3)	N5—C22—C23	115.3 (3)
C4—C3—N1	117.4 (3)	C21—C22—C23	123.3 (3)
C2—C3—N1	119.0 (3)	N6—C23—C24	121.2 (3)
C3—C4—C5	119.0 (3)	N6—C23—C22	115.9 (2)
C3—C4—H4A	120.5	C24—C23—C22	122.8 (3)
C5—C4—H4A	120.5	C25—C24—C23	119.2 (4)
C4—C5—C6	120.4 (3)	C25—C24—H24A	120.4
C4—C5—N2	120.0 (3)	C23—C24—H24A	120.4
C6—C5—N2	119.5 (3)	C24—C25—C26	119.5 (3)
C1—C6—C5	121.3 (3)	C24—C25—H25A	120.2
C1—C6—H6A	119.4	C26—C25—H25A	120.2
C5—C6—H6A	119.4	C25—C26—C27	118.6 (4)
O1—C7—O2	124.2 (3)	C25—C26—H26A	120.7
O1—C7—C1	118.0 (3)	C27—C26—H26A	120.7
O2—C7—C1	117.8 (3)	N6—C27—C26	122.4 (4)
N3—C8—C9	123.4 (4)	N6—C27—H27A	118.8
N3—C8—H8A	118.3	C26—C27—H27A	118.8
			
O3—Co1—N3—C8	−100.0 (3)	C2—C1—C6—C5	−2.1 (5)
N5—Co1—N3—C8	−4.6 (3)	C7—C1—C6—C5	176.5 (3)
N4—Co1—N3—C8	173.0 (3)	C4—C5—C6—C1	1.7 (5)
N6—Co1—N3—C8	71.8 (3)	N2—C5—C6—C1	−177.3 (3)
O3—Co1—N3—C12	89.3 (2)	Co1—O2—C7—O1	149.7 (3)
N5—Co1—N3—C12	−175.2 (2)	Co1—O2—C7—C1	−32.9 (4)
N4—Co1—N3—C12	2.4 (2)	C6—C1—C7—O1	35.5 (5)
N6—Co1—N3—C12	−98.9 (2)	C2—C1—C7—O1	−145.9 (4)
O2—Co1—N4—C17	−7.7 (2)	C6—C1—C7—O2	−142.0 (3)
O3—Co1—N4—C17	81.0 (2)	C2—C1—C7—O2	36.6 (5)
N3—Co1—N4—C17	175.4 (3)	C12—N3—C8—C9	1.2 (5)
N6—Co1—N4—C17	−100.2 (2)	Co1—N3—C8—C9	−169.4 (3)
O2—Co1—N4—C13	−179.9 (2)	N3—C8—C9—C10	−1.3 (6)
O3—Co1—N4—C13	−91.2 (2)	C8—C9—C10—C11	0.2 (5)
N3—Co1—N4—C13	3.2 (2)	C9—C10—C11—C12	1.0 (5)
N6—Co1—N4—C13	87.7 (2)	C8—N3—C12—C11	0.1 (4)
O2—Co1—N5—C18	88.5 (3)	Co1—N3—C12—C11	171.4 (2)
O3—Co1—N5—C18	−0.4 (3)	C8—N3—C12—C13	−178.3 (3)
N3—Co1—N5—C18	−94.8 (3)	Co1—N3—C12—C13	−7.0 (3)
N6—Co1—N5—C18	179.7 (3)	C10—C11—C12—N3	−1.1 (5)
O2—Co1—N5—C22	−88.8 (2)	C10—C11—C12—C13	177.1 (3)
O3—Co1—N5—C22	−177.7 (2)	C17—N4—C13—C14	0.4 (4)
N3—Co1—N5—C22	87.9 (2)	Co1—N4—C13—C14	173.0 (2)
N6—Co1—N5—C22	2.4 (2)	C17—N4—C13—C12	179.6 (3)
O2—Co1—N6—C27	−90.8 (3)	Co1—N4—C13—C12	−7.8 (3)
N5—Co1—N6—C27	179.4 (3)	N3—C12—C13—N4	9.8 (4)
N3—Co1—N6—C27	81.9 (3)	C11—C12—C13—N4	−168.6 (3)
N4—Co1—N6—C27	6.5 (3)	N3—C12—C13—C14	−171.1 (3)
O2—Co1—N6—C23	86.5 (2)	C11—C12—C13—C14	10.5 (5)
N5—Co1—N6—C23	−3.3 (2)	N4—C13—C14—C15	0.3 (5)
N3—Co1—N6—C23	−100.8 (2)	C12—C13—C14—C15	−178.8 (3)
N4—Co1—N6—C23	−176.2 (2)	C13—C14—C15—C16	0.2 (6)
O3—Co1—O2—C7	3.1 (3)	C14—C15—C16—C17	−1.2 (7)
N5—Co1—O2—C7	−91.9 (3)	C13—N4—C17—C16	−1.6 (5)
N4—Co1—O2—C7	91.0 (3)	Co1—N4—C17—C16	−173.6 (3)
N6—Co1—O2—C7	−168.6 (3)	C15—C16—C17—N4	2.0 (6)
O2—Co1—O3—C2	32.9 (2)	C22—N5—C18—C19	−0.6 (5)
N5—Co1—O3—C2	122.9 (2)	Co1—N5—C18—C19	−177.9 (3)
N3—Co1—O3—C2	−140.2 (2)	N5—C18—C19—C20	0.2 (6)
N4—Co1—O3—C2	−64.2 (3)	C18—C19—C20—C21	0.1 (6)
Co1—O3—C2—C1	−38.2 (4)	C19—C20—C21—C22	0.0 (6)
Co1—O3—C2—C3	141.4 (2)	C18—N5—C22—C21	0.7 (5)
C6—C1—C2—O3	−179.7 (3)	Co1—N5—C22—C21	178.2 (3)
C7—C1—C2—O3	1.8 (5)	C18—N5—C22—C23	−178.7 (3)
C6—C1—C2—C3	0.7 (4)	Co1—N5—C22—C23	−1.2 (4)
C7—C1—C2—C3	−177.8 (3)	C20—C21—C22—N5	−0.4 (5)
O3—C2—C3—C4	−178.6 (3)	C20—C21—C22—C23	178.9 (3)
C1—C2—C3—C4	1.0 (4)	C27—N6—C23—C24	1.8 (5)
O3—C2—C3—N1	−3.2 (4)	Co1—N6—C23—C24	−175.7 (3)
C1—C2—C3—N1	176.4 (3)	C27—N6—C23—C22	−178.7 (3)
O4—N1—C3—C4	−144.3 (3)	Co1—N6—C23—C22	3.8 (4)
O5—N1—C3—C4	32.0 (4)	N5—C22—C23—N6	−1.8 (4)
O4—N1—C3—C2	39.9 (4)	C21—C22—C23—N6	178.8 (3)
O5—N1—C3—C2	−143.7 (3)	N5—C22—C23—C24	177.7 (3)
C2—C3—C4—C5	−1.3 (5)	C21—C22—C23—C24	−1.7 (5)
N1—C3—C4—C5	−176.9 (3)	N6—C23—C24—C25	−0.7 (6)
C3—C4—C5—C6	0.0 (5)	C22—C23—C24—C25	179.9 (4)
C3—C4—C5—N2	179.0 (3)	C23—C24—C25—C26	−1.2 (6)
O6—N2—C5—C4	−1.7 (5)	C24—C25—C26—C27	1.8 (7)
O7—N2—C5—C4	179.5 (3)	C23—N6—C27—C26	−1.1 (5)
O6—N2—C5—C6	177.4 (3)	Co1—N6—C27—C26	176.1 (3)
O7—N2—C5—C6	−1.4 (5)	C25—C26—C27—N6	−0.7 (6)

### Hydrogen-bond geometry (Å, °)

**Table:**

*D*—H···*A*	*D*—H	H···*A*	*D*···*A*	*D*—H···*A*
C11—H11A···O1^i^	0.93	2.32	3.166 (5)	151
C21—H21A···O1^ii^	0.93	2.40	3.275 (4)	157
C16—H16A···O4^iii^	0.93	2.43	3.171 (5)	137
C25—H25A···O6^iv^	0.93	2.61	3.209 (5)	123
C9—H9A···O5^v^	0.93	2.61	3.346 (5)	136

Symmetry codes: (i) −*x*+3/2, *y*−1/2, −*z*+3/2; (ii) −*x*+1, −*y*+1, −*z*+2; (iii) *x*+1, *y*, *z*; (iv) *x*, *y*, *z*+1; (v) *x*−1/2, −*y*+1/2, *z*+1/2.

## References

[bb1] Lemoine, P., Viossat, B., Dung, N. H., Tomas, A., Morgant, G., Greenaway, F. T. & Sorenson, J. R. J. (2004). *J. Inorg. Biochem.* **98**, 1734–1749. 10.1016/j.jinorgbio.2004.07.010 15522401

[bb2] Rigaku (1998). *PROCESS-AUTO* Rigaku Corporation, Tokyo, Japan.

[bb3] Sheldrick, G. M. (2008). *Acta Cryst.* A**64**, 112–122. 10.1107/S0108767307043930 18156677

[bb4] Wen, D.-C. & Liu, S.-X. (2007). *Chin. J. Struct. Chem.* **11**, 1360–1366.

[bb5] Wen, D.-C., Liu, S.-X. & Lin, M. (2007*a*). *J. Mol. Struct.* **876**, 154–161.

[bb6] Wen, D.-C., Liu, S.-X. & Ribas, J. (2007*b*). *Inorg. Chem. Commun.* **10**, 661–665.

[bb7] Wen, D.-C., Liu, S.-X. & Ribas, J. (2007*c*). *Polyhedron*, **26**, 3849–3856.

[bb8] Wen, D.-C., Wu, L.-H., Zhong, C.-L., Xie, T.-Y. & Ta, H.-G. (2007*d*). *Acta Cryst.* E**63**, m2362–m2363.

[bb9] Wen, D.-C. & Xie, T.-Y. (2007). *Inorg. Chem. Commun.* **10**, 1531–1533.

[bb10] Yin, M.-C., Yuan, L.-J., Ai, C.-C., Wang, C.-W., Yuan, E.-T. & Sun, J.-T. (2004). *Polyhedron*, **23**, 529–536.

